# Spatiotemporal and Kinetic Determinants of Sprint Acceleration Performance in Soccer Players

**DOI:** 10.3390/sports6040169

**Published:** 2018-12-09

**Authors:** Munenori Murata, Yohei Takai, Hiroaki Kanehisa, Tetsuo Fukunaga, Ryu Nagahara

**Affiliations:** National Institute of Fitness and Sports in Kanoya, Kanoya 891-2393, Japan; mmurata@nifs-k.ac.jp (M.M.); y-takai@nifs-k.ac.jp (Y.T.); hkane@nifs-k.ac.jp (H.K.); fukunaga@nifs-k.ac.jp (T.F.)

**Keywords:** ground reaction force, running speed, step length, step frequency, football

## Abstract

We aimed to elucidate spatiotemporal and kinetic determinants of sprint acceleration performance in soccer players. Thirty-seven male soccer players performed 60-m sprints. The spatiotemporal variables and ground reaction impulses were calculated over a 50-m distance. When controlling the influence of stature and body mass, change in running speed was correlated with the step length at the 1st–4th step section (r = 0.695), step frequency from the 9th to 20th step sections (r = 0.428 to 0.484), braking impulse during the 17th–20th step section (r = 0.328), propulsive impulse from the 1st to 8th step sections (r = 0.738 and 0.379), net anteroposterior impulse for all step sections (r = 0.384 to 0.678), and vertical impulse from the 9th–12th step section and thereafter (r = −0.355 to −0.428). These results confirmed that an effective acceleration is probably accomplished by a greater step length originated in greater propulsive impulse during the initial acceleration phase (to the 8th step), a higher step frequency through smaller vertical impulse and smaller braking impulse during the middle and later acceleration phases (from the 9th step), as well as greater net anteroposterior impulse during the entire acceleration phase.

## 1. Introduction

Sprint performance, especially acceleration performance, is decisive for attacking play toward the goal in a soccer game [1,2]. Thus, improving sprint acceleration ability is of great importance for soccer players to beat opposition competitors [3]. For this reason, many studies have monitored sprint performance of soccer players during training and match games [4,5,6]. For soccer players, however, available information concerning determinants of sprint acceleration performance is scarce.

From the findings of a prior study, which investigated the relationship between sprint performance and spatiotemporal variables for soccer players [7], the running speed was correlated with step length (SL) during the initial and middle acceleration phases (until the 16th step). However, a previous study, which examined the difference between faster and slower field sport athlete groups including soccer players, reported that the faster group was accompanied by greater step frequency (SF) during the initial three steps [8]. Moreover, for rugby players, it was verified that SF was higher in backs (faster) group than forwards (slower) group while SL showed moderate relationship with sprinting performance within forwards players during the initial three steps [9]. These conflicting findings in the previous studies partially resulted from the difference in variables used, in addition to differences in performance level and characteristics of athletes. So as to correctly understand the determinants of sprint acceleration performance, it is likely better to adopt changes in running speed (*∆v*) or acceleration as a performance indicator. However, no studies using field athlete groups have adopted *∆v* or acceleration as a dependent variable. Evaluating the associations of increment of running speed or acceleration with either SL or SF would improve our understanding of the sprint acceleration performance in soccer players.

The *∆v* mechanically depend on ground reaction forces (GRFs). Thus, investigating GRF in addition to spatiotemporal variables can provide a better understanding of the source of effective sprint acceleration through an increase in SL or SF. For soccer players, however, previous studies have not investigated GRFs during the acceleration phase of a single maximal sprint. Earlier studies employing sprinters have shown that, for better sprint acceleration, while greater propulsive force was a prominent factor during the entire acceleration phase [10,11], a smaller braking force was also a determinant when the running speed was greater than or equal to 70% of the maximum [10]. Although these findings are possibly useful for soccer players, whether the previous findings are applicable for soccer players is unclear. The majority of sprint distances for soccer players during a match are shorter than 20 m [3]. In addition, soccer players often initiate their sprints from a standing posture during a match. Thus, it may be assumed that the kinetic determinants of sprint acceleration performance for soccer players would differ from those for sprinters. Clarifying this aspect will be useful for understanding the determinants of better sprinting performance in soccer players and for designing training modalities aiming to improve them.

The purpose of this study was, therefore, to elucidate the determinants of sprint acceleration performance in soccer players on the basis of the associations of the *∆v* with spatiotemporal and kinetic variables during the sprinting. We hypothesized that longer SL and greater propulsive force in the initial acceleration section would be associated with greater acceleration.

## 2. Materials and Methods

### 2.1. Participants

Thirty-seven male soccer players (field players), who belonged to a university team (mean ± SD: age, 19.9 ± 1.2 year; stature, 1.71 ± 0.06 m; body mass, 66.0 ± 6.2 kg), participated in this study. This study was approved beforehand by the research ethics committee of the institute. The aims, risks of involvement, and experimental conditions of the study were explained before participation. Written informed consent was then obtained from all participants.

### 2.2. Experimental Procedures

The participants performed two maximal effort 60-m sprints from a crouched split standing position. A rest period was 10 min between the trials. GRFs for a 50-m distance were recorded using fifty force platforms (1.0 × 0.9 m; TF-90100, Tec Gihan, Uji, Japan; 1000 Hz) (see Nagahara et al. [7,12] for details).

### 2.3. Data Processing

Spatiotemporal variables at each step over the 50-m distance were calculated from GRF data. Foot strike and toe-off instants were detected with a threshold of vertical GRF being over 20 N. Support time (ST) was the duration while the foot was touching the ground, and aerial time (AT) was the duration while neither foot was touching the ground. SF was computed as the inverse of the sum of ST and AT at each step. Foot location during the support phase was obtained as the mean of the anteroposterior center of pressure position for 0.01 s (10 data points) during the middle of the support phase [10]. SL was determined as the distance between two consecutive foot locations in the anteroposterior direction. Running speed was calculated as a product of SL and SF. Step-to-step braking (*Bimp*), propulsive (*Pimp*) and vertical impulses (*Vimp*) over the 50-m distance were computed integrating respective forces during the corresponding durations using a trapezoid formula. Net anteroposterior impulse (*APimp*) was calculated integrating the anteroposterior force during the entire support phase. All impulse variables were expressed as the values relative to body mass. Based on a mean running speed for the 50-m distance, the fastest trial from each participant was adopted for the following data processing.

### 2.4. Statistical Analyses

To cancel the bilateral difference and influence of human cyclic movement variability, average values for every four steps were calculated. The average maximal speed appeared at the 21st step, therefore, five values during accelerated sprinting until the 20th step (1st–4th, 5th–8th, 9th–12th, 13th–16th, and 17th–20th step sections) were obtained. Then, the difference in running speed between adjacent sections (except for 1st–4th section) was computed as *∆v*. Descriptive data of the computed variables were expressed as means and standard deviations. The Shapiro–Wilk test was used to test normality of the data. Then, partial correlation coefficients with control variables of stature and body mass were calculated to examine the relationships of *∆v* with the spatiotemporal and impulse variables without the influence of body size on them. The significance level was set at *P* < 0.05.

## 3. Results

**Figure 1:**
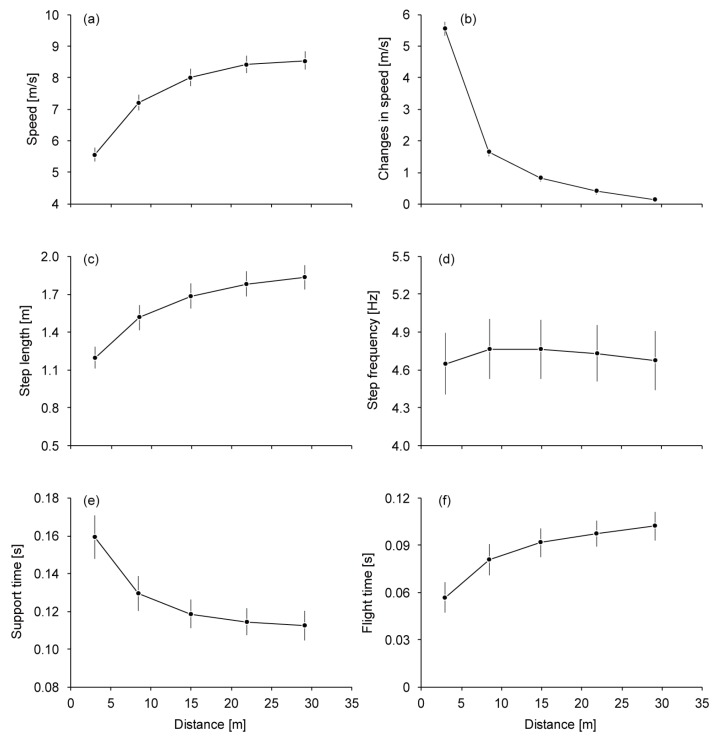
Changes in spatiotemporal variables during sprint acceleration. (**a**) Running speed, (**b**) change in running speed, (**c**) step length, (**d**) step frequency, (**e**) support time and (**f**) aerial time. Mean values are plotted at each step section. The vertical lines show standard deviations.

**Figure 2:**
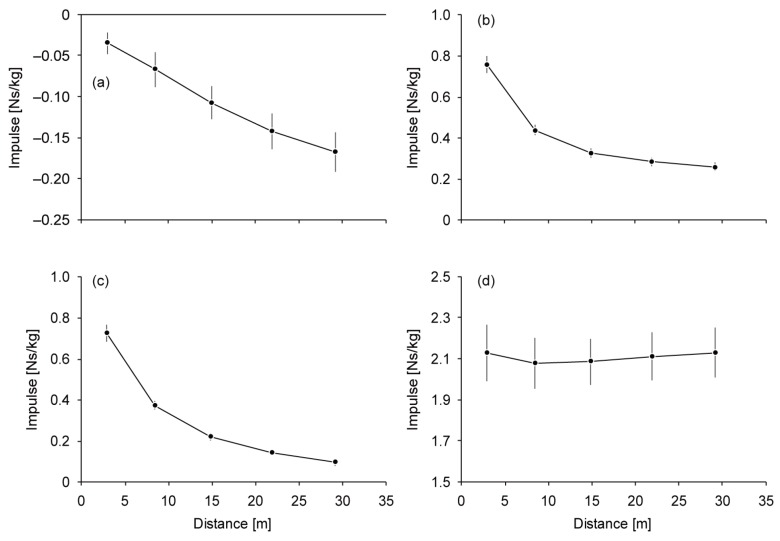
Changes in impulse variables during sprint acceleration. (**a**) Braking impulse, (**b**) propulsive impulse, (**c**) net anteroposterior impulse and (**d**) vertical impulse. Mean values are plotted at each step section. The vertical lines show standard deviations.

The mean running speed over the 50-m distance in the faster trial was 7.91 ± 0.24 m/s. Running speed and SL increased until the 17th–20th step section, reaching 8.54 ± 0.28 m/s and 1.83 ± 0.10 m, respectively (Figure 1a,c). The *∆v* decreased from 5.56 ± 0.22 m/s to 0.13 ± 0.08 m/s through the entire acceleration phase (Figure 1b). SF increased to the 5th–8th step section (4.77 ± 0.23 Hz) and then slightly decreased to the 17th–20th step section (Figure 1d). ST and AT decreased for 0.047 ± 0.009 s and increased for 0.045 ± 0.010 s, respectively, until the 17th–20th step section (Figure 1e,f). While *Bimp* increased until the 17th–20th step section (−0.17 ± 0.02 Ns/kg) (Figure 2a), *Pimp* (0.76 ± 0.04 Ns/kg at the 1st–4th step section) and *APimp* (0.72 ± 0.04 Ns/kg at the 1st–4th step section) decreased through the entire acceleration phase (Figure 2b,c). *Vimp* decreased to the 5th–8th step section and increased slightly afterward (2.13 ± 0.12 Ns/kg) (Figure 2d).

**Figure 3:**
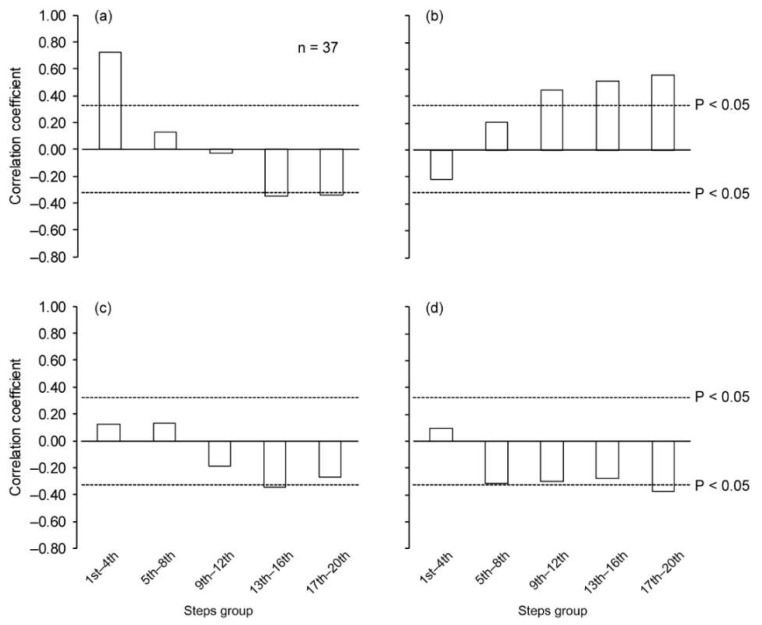
Partial correlation coefficient of change in running speed with spatiotemporal variables at each step section, controlling for stature and body mass. (**a**) Step length, (**b**) step frequency, (**c**) support time and (**d**) aerial time. Dotted horizontal lines indicate correlation coefficient at *P* = 0.05.

**Figure 4:**
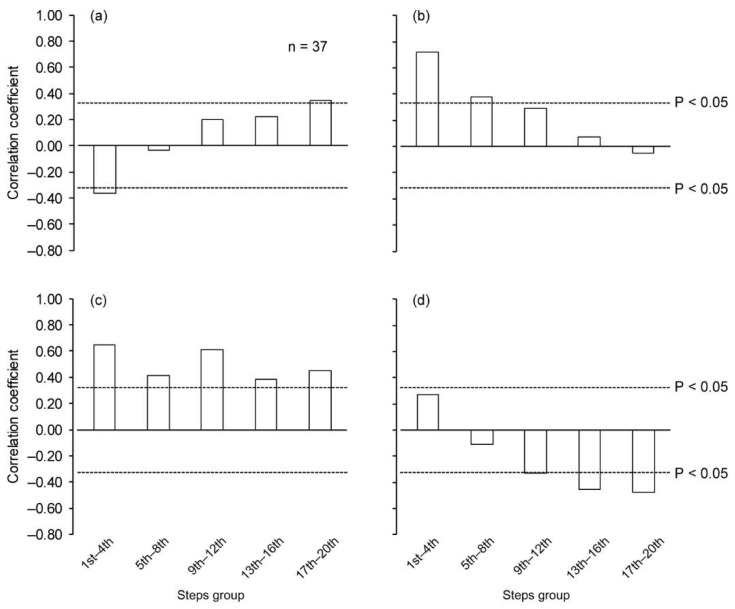
Partial correlation coefficient of change in running speed with impulse variables at each step section, controlling stature and body mass. (**a**) Braking impulse, (**b**) propulsive impulse, (**c**) net anteroposterior impulse and (**d**) vertical impulse. Dotted horizontal lines indicate correlation coefficient at *P* = 0.05.

When controlling for the influence of stature and body mass, the *∆v* was significantly correlated with the SL positively at the 1st–4th step section and negatively at the 13th–16th and 17th–20th step sections (Figure 3a), and those in SF positively from the 9th–12th to the 17th–20th step section (Figure 3b). Significant negative correlations for *∆v* versus ST and AT were found at the 13th–16th and 17th–20th step sections, respectively (Figure 3c,d). The *∆v* was significantly correlated with *Bimp* during the first and last step sections (Figure 4a). There were significant positive correlations between *∆v* and Pimp for the initial two step sections (Figure 4b). For the *APimp*, there were significant correlations with *∆v* for all step sections (Figure 4c). Significant negative correlations were found for *∆v* versus *Vimp* from the 9th–12th step section and thereafter (Figure 4d).

## 4. Discussion

This study aimed to elucidate spatiotemporal and kinetic determinants of sprint acceleration in soccer players. The current findings indicate that a greater SL during the initial acceleration phase (1st–4th step section), a higher SF during the middle and later acceleration phases (from the 9th to 20th step), and shorter ST and AT during the later acceleration phase (13th–16th and 17th–20th step sections) are essential for greater sprint acceleration. Moreover, for better sprint acceleration performance, smaller *Bimp* during the later acceleration phase (17th–20th step section), greater Pimp during initial two step sections (from the 1st to 8th step), a greater *APimp* during the entire acceleration phase, and smaller *Vimp* during the middle and later acceleration phases (from the 9th to 20th step) were found to be important. Overall, the findings in this study supported our hypotheses.

While previous studies showed contradiction about the determinants of the initial sprint acceleration performance of field sport athletes in terms of SL and SF [7,8,9], the only study employed soccer players elucidated that the greater running speed was associated with greater SL [7]. The results in this study support the previous finding [7] and indicates the importance of SL for greater increment of running speed during the initial acceleration phase in soccer players. Although the running speed was associated with SF in previous studies using field sport athletes [8,9], magnitude of SF was relatively high in the current study compared to those in the previous studies (4.65 ± 0.24 Hz in the 1st–4th step section in this study versus 3.64 and 3.34 Hz in the initial three steps in the study by Murphy et al. [8] and ranged from 4.0 to 4.6 Hz during the initial three steps in the study by Wild et al. [9]). These indicate that baseline of SF was high during the initial acceleration phase in this study, and this high SF would limit the variation of SF possibly mitigating the association of SF with increment of running speed. From the 9th–12th to the 17th–20th step section, SF was correlated with *∆v*, indicating that higher SF is essential for the greater increment of running speed during the middle and later acceleration phases. The fact that ST and AT were negatively correlated with *∆v* at the 13th–16th and 17th–20th step sections demonstrates the importance of shorter ST and AT for better acceleration during the later acceleration phase. Although the current result cannot be compared to those of previous studies using soccer players or field sport athletes, a study employed sprinters also found the importance of greater decrease in ST and suppressing the increment of AT for greater acceleration through higher SF during the later acceleration phase in maximal sprinting [13]. Thus, the finding in this study is in line with the previous study using sprinters.

The *APimp* was associated with *∆v* at all step sections. This indicates that the amount of forward oriented GRF is a predominant factor for better sprint acceleration in soccer players, consisting with previous findings obtained in sprinters [10,11,14]. For sub-components, greater *Pimp* and smaller *Bimp* were associated with greater *∆v* during the 1st–4th to 5th–8th step section and the 17th–20th step section, respectively. These results demonstrate that producing greater propulsive force during the initial acceleration phase and suppressing braking force during the later acceleration phase are essential for better acceleration performance. Although there is no study that investigated the relationship of *Pimp* and *Bimp* with sprinting performance using soccer players, the current findings are consistent to previous findings using sprinters [10]. The *∆v* was negatively correlated with the *Vimp*. This conflicts to a previous finding which showed no association of running speed with *Vimp* in soccer players during the entire acceleration phase [7]. The contradiction may be due to the difference in the used variables (*∆v* in this study versus running speed in the previous study). Moreover, the current finding is in line with a previous study which examined the relationship of running acceleration with mean vertical force in sprinters during accelerated sprinting [10]. In addition, this smaller *Vimp* probably resulted in higher SF through shorter AT during the middle and later acceleration phases. Taken together, it can be said that the smaller *Vimp* is of importance for better sprint acceleration especially during the middle and later acceleration phases. The current results indicate that greater *Bimp* possibly relate to greater *∆v* during the 1st–4th step section. However, the braking force and impulse during the initial acceleration phase were very small [10], and thus it may not be important to produce braking force during the initial acceleration phase for better acceleration.

Before summarizing the current results, the reader should be aware of several limitations in this study. The sprint distance was set to 60-m in order to cover the entire acceleration phase of sprinting for all participants [10,13]. However, the vast majority of sprints in a soccer game are shorter than 20-m [3]. In addition, while this study adopted the crouched split standing start as used in previous studies using soccer players [3,6,7,15,16], soccer players often initiate sprints from a jogging or nonstationary condition in their own competitive games. Thus, there is a possibility that the determinants of sprint acceleration performance in soccer players could be different from those suggested by the findings obtained here. Further study is needed to elucidate the influences of the running distance and conditions prior to the start of the accelerated sprint on the determinants of sprint acceleration performance in soccer players. The participants used in this study were collegiate soccer players. Thus, it is possible that the results might differ when professional soccer players are employed, and further study including professional players is needed to generalize the current findings.

## 5. Conclusions

The results in this study confirmed that an effective acceleration is probably accompanied by a greater step length through greater propulsive impulse during the initial acceleration phase (up to the 8th step), a higher step frequency through shorter support and aerial times and smaller vertical impulse, and smaller braking impulse during the middle and later acceleration phases (from the 9th to 20th step), as well as greater net anteroposterior impulse during the entire acceleration phase. The findings in this study will be useful for considering sprint training for soccer players.
